# Supplementary material for: Application of Amplicon-Based Targeted NGS Technology for Diagnosis of Drug-Resistant Tuberculosis Using FFPE Specimens
Source: Microbiol Spectr. 2022 Feb 9;10(1):e01358-21. doi: 10.1128/spectrum.01358-21 (PMC8826733; doi:10.1128/spectrum.01358-21)
Supplement: SUPPLEMENTAL FILE 4 — Supplemental material. Download SPECTRUM01358-21_Supp_1_seq1.pdf, PDF file, 0.2 MB [file spectrum01358-21_supp_1_seq1.pdf]

Supplementary Table1 List of drug-resistant genes and mutation sites in the amplicon-based targeted NGS panel

Drug	Genes	Mutation sites
Rifampin	rpoB	Q432K/L/P, L430P, D435V/A/F/G/N/Y, S450L/F, H445C/D/F/G/L/N/P/R/Y/S, L452P
Isoniazid	KatG<br>inhA-fabG promoter<br>inhA	S315T, S315N<br>C-8t, c-15t<br>I21T, S94A, I194T
Ethambutol	embB	M306V/I, Q497K/R, D354A, G406S/D/A
Streptomycin	rpsL<br>rrs	K43R, K88R/Q<br>a514c, c517t
Kanamycin	rrs<br>eis promoter	a1401g, c1402t<br>g-10a, c-14t, g-37t
Amikacin	rrs	a1401g, c1402t
Capreomycin	rrs<br>tlyA	a1401g, c1402t<br>N236K
Fluoroquinolone	gyrA<br>gyrB	G88A/C, S91P, A90V, D94A/G/H/N/Y<br>A504V
